# A Novel Sucrose-Regulatory MADS-Box Transcription Factor GmNMHC5 Promotes Root Development and Nodulation in Soybean (*Glycine max* [L.] Merr.)

**DOI:** 10.3390/ijms160920657

**Published:** 2015-08-31

**Authors:** Wei Liu, Xiangdong Han, Ge Zhan, Zhenfang Zhao, Yongjun Feng, Cunxiang Wu

**Affiliations:** 1 The National Key Facility for Crop Gene Resources and Genetic Improvement and MOA Key Lab of Soybean Biology (Beijing), Institute of Crop Sciences, the Chinese Academy of Agricultural Sciences, 12 Zhongguancun South Street, Haidian District, Beijing 100081, China; E-Mails: hnaulw@126.com (W.L.); hxdtjnu@126.com (X.H.); zhan_ge_edu@sina.com (G.Z.); zhaozf2009@126.com (Z.Z.); 2 School of Life Science, Beijing Institute of Technology, 5 Zhongguancun South Street, Haidian District, Beijing 100081, China

**Keywords:** *Glycine max*, GmNMHC5, lateral roots development, MADS-box protein, sucrose, nodule building

## Abstract

The MADS-box protein family includes many transcription factors that have a conserved DNA-binding MADS-box domain. The proteins in this family were originally recognized to play prominent roles in floral development. Recent findings, especially with regard to the regulatory roles of the AGL17 subfamily in root development, have greatly broadened their known functions. In this study, a gene from soybean (*Glycine max* [L.] Merr.), *GmNMHC5*, was cloned from the Zigongdongdou cultivar and identified as a member of the *AGL17* subfamily. Real-time fluorescence quantitative PCR analysis showed that *GmNMHC5* was expressed at much higher levels in roots and nodules than in other organs. The activation of expression was first examined in leaves and roots, followed by shoot apexes. *GmNMHC5* expression levels rose sharply when the plants were treated under short-day conditions (SD) and started to pod, whereas low levels were maintained in non-podding plants under long-day conditions (LD). Furthermore, overexpression of *GmNMHC5* in transgenic soybean significantly promoted lateral root development and nodule building. Moreover, GmNMHC5 is upregulated by exogenous sucrose. These results indicate that GmNMHC5 can sense the sucrose signal and plays significant roles in lateral root development and nodule building.

## 1. Introduction

Soybean (*Glycine max* [L.] Merr.) is one of the most important oil and high-protein food/forage crops in the world. A prominent feature of soybean growth is that the staggered time of vegetative growth and reproductive development is much longer than many other crops, such as grains. For example, the overlapped time in cultivar Zigongdongdou accounts for approximately 40% or even longer of the entire growth period [1]. During that time, there exists fierce competition in the material and energy demands between the reproductive organs and vegetative organs, with that between flowering and nodulation being the most representative [1]. The process of flowering occurs in the aerial parts, whereas nodulation occurs in the underground parts; therefore, the photosynthate produced in the leaves must be appropriately allocated to these two (upper and lower) extremes to ensure that the two processes are systematically performed. Therefore, the elucidation of the bifunctional regulation in both flowering and nodulation (or root development) is valuable for understanding the cooperative associations of the two processes in soybean.

MADS-box transcription factors play important roles in diverse developmental processes in flowering plants. Initially, studies on the function of MADS-box genes were focused on flowering. Later, an increasing number of members and features of MADS-box genes were discovered. The Arabidopsis MADS-box genes, e.g., *AGAMOUS-LIKE 20* (*AGL20*), *SUPPRESSOR OF OVEREXPRESSION OF CONSTANS 1* (*SOC1*) and *AGAMOUS-LIKE 28* (*AGL28*), positively regulate the flowering process through one or more such pathways [2,3,4]. In turn, *FLOWERING LOCUS C* (*FLC*), *AGAMOUS-LIKE 18* (*AGL18*), and *SHORT VEGETATIVE PHASE* (*SVP*) negatively regulate flowering [5,6,7].

Strikingly, some MADS-box genes were found to be involved in the regulation of root development. The *AGL17* clade gene *ARABIDOPSIS NITRATE REGULATED 1* (*ANR1*), a key determinant of developmental plasticity in Arabidopsis roots, has a function in nutrient response in the roots and controls lateral root elongation in response to nitrate [8]. Other genes in the *AGL17* clade, such as *AGL17* and *AGL21*, were reported to be highly expressed in Arabidopsis roots, indicating their potential roles in root development [9].

In fact, a number of MADS-box transcription factors have multiple functions in plant development. The *AGL17* clade gene *AGL17* was detectable in various plant organs yet with the highest expression in the root, indicating a potential role in root development [9]. Nevertheless, *AGL17* was also observed to promote flowering, which is positively controlled by the photoperiod pathway regulator CONSTANS CO [10]; *AGL12* is another important regulator in root development and also a promoter of the floral transition [11]; another *AGL17-like* gene, *AGL16* functions in the satellite meristemoid lineage of stomatal development and flowering transition [12,13]. AG-like genes *SHATTERPROOF 1*, *2* (*SHP1*, *2*) and *SEEDSTICK* (*STK*) are both involved in the flower development and periodic lateral root formation [14,15]. In addition, a AGL6-like gene AGL6 regulates flowering transition and lateral organ development [16,17].

The genes *nmh7*, *nmhC5* and *ngl9* were initially identified from the nodules of *Medicago sativa* [18,19,20]*.* An evolutionary analysis among MADS-box family members showed that *nmh7* and *ngl9* were orthologous to the floral subfamily represented by AP3 (DefA)/PI (Glo) [18].The presence of NMH7 in non-inoculated seeds, cotyledon seedling and primary roots suggests that this protein might be involved in non-symbiotic events or that NMH7 might be involved in nodule developmental programs related to bacteria colonization [21]. The functions of NGL9 and NMH7 depend on their binding as a heterodimer [20]. A glycolytic enzyme, Fructose-1,6-bisphosphatealdolase cytosolic class I (aldolase) was identified as a putative binding NMH7 partner [22]. Our previous work demonstrated that *GmNMH7* from *G. max* might be involved in both flower and nodule development [23]. NMHC5, being orthologous to root-expressed *AGL17* subfamily proteins in *Arabidopsis thaliana*, forms homodimers and performs its functions by binding to a CArG consensus sequence *in vitro* [18]. The characteristic of formation of a homodimer formation by in NMHC5 alone also benefits the studies from a simplified operation, in contrast to dealing with NGL9 and NMH7 simultaneously to obtain a functional heterodimer. However, the function of NMHC5 has not yet been described. In this study, a MADS-box gene named as GmNMHC5, homologous to *NMHC5* was cloned from *G. max*. Transcription of *GmNMHC5* was accumulated in roots and nodules and up regulated by sucrose; its overexpression affected the lateral root growth and nodulation, and expression pattern in different tissues also suggest the potential roles of GmNMHC5 in soybean flowering and pod formation.

## 2. Results

### 2.1. Gene Cloning and Phylogenic Analysis of the GmNMHC5 Protein

According to the results of a BLAST search with the sequence of *NMHC5* from *Medicago sativa* in the soybean protein sequence database in Phytozome 8.9, a protein orthologous to MsNMHC5, mapped to chromosome 13 of *Glycine max*, was obtained. The full-length CDS (726-bp) of the target protein in soybean cultivar Zigongdongdou was then obtained by RT-PCR using RNA extracted from nodules.

**Figure 1:**
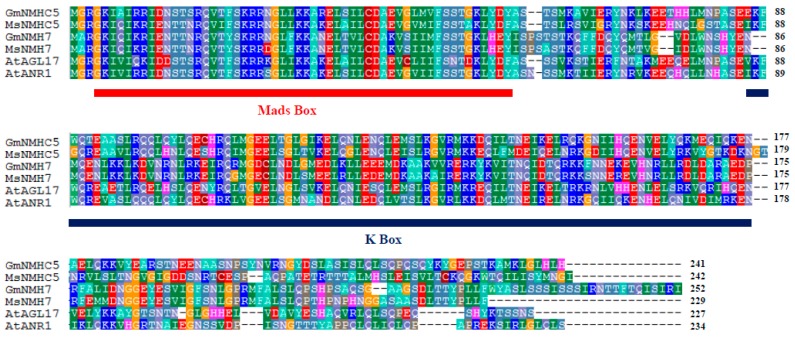
Sequence alignment between GmNMHC5 and other related MADS-box proteins (Gm, *G. max*; At, *Arabidopsis thaliana*; Ms, *Medicago sativa*); the MADS-box and K-box domains are indicated by red and blue lines, respectively. Accession numbers are as flollows: GmNMHC5 (NP_001241489.1), MsNMHC5 (AAB51377.1), GmNMH7 (NP_001236857.1), MsNMH7 (AEW43601.1), AtAGL17 (NP_179848.1), AtANR1 (CAB09793.1).

*GmNMHC5* encodes a protein sequence of 241 amino acids that shares high homology to the alfalfa MsNMHC5 protein and other root-specific expressed gene products (Figure 1). GmNMHC5 was predicted to share 52% identity with MsNMHC5 and MsNMH7, 62.4% identity with AtANR1, 57.85% identity with AtAGL17. All sequences contain the MEF2 (myocyte enhancer factor 2)-type MADS-box and a K-box, sharing a high similarity in these two conserved regions. Therefore, GmNMHC5 is an MIKC-type MADS-box transcription factor.

**Figure 2:**
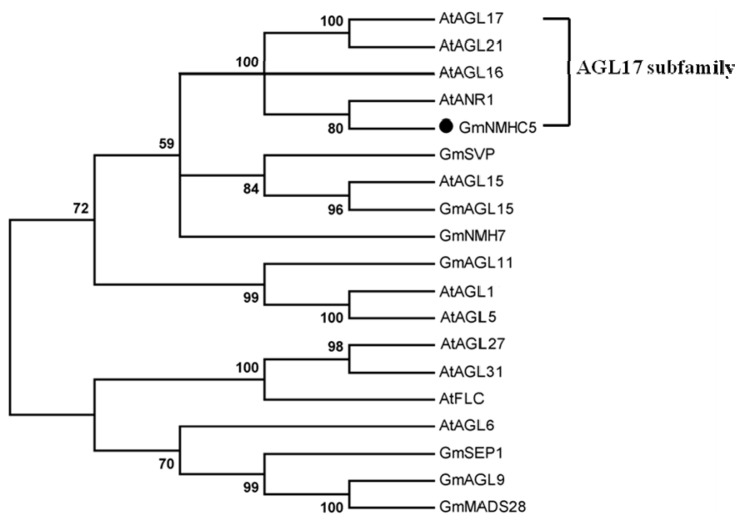
Phylogenetic tree based on protein sequences between GmNMHC5 and some other function-known MIKC-type MADS-box transcription factors of *Glycine max* and *Arabidopsis thaliana* (Gm, *G. max*; At, *Arabidopsis thaliana*). Accession numbers are as follows: AtAGL17 (NP_179848.1), AtAGL21 (NP_195507.1), AtAGL16 (NP_191282.2), AtANR1 (CAB09793.1), GmNMHC5 (NP_001241489.1), GmSVP (ACJ61500.1), AtAGL15 (NP_196883.1), GmAGL15 (NP_001237033.1), GmNMH7 (NP_001236857.1), GmAGL11 (NP_001236130.1), GmAGL1 (NP_191437.1), GmAGL5 (NP_565986.1), GmAGL27 (NP_177833.3), AtAGL31 (NP 001119498.1), AtFLC (NP_196576.1), AtAGL6 (NP_182089.1), GmSEP1 (NP_001238296.1), GmAGL9 (ACA24481.1), GmMADS28 (NP 001236390.1). The Phylogenetic tree was constructed using the Maximum Likelihood method of phylogenetic tree construction, with 200 bootstrap replicates, using MEGA v.5.05. The number for each node is the bootstrap percentages, and nodes with less than 70% bootstrap values were collapsed.

A phylogenetic analysis of the amino acid sequence with several reported members of the MIKC-type MADS-box transcription factors from *Glycine max* and *Arabidopsis thaliana* was conducted; as shown in the result, GmNMHC5 was closely related to the AGL17 [24] subfamily (Figure 2).

A bioinformatic analysis on the region upstream of *GmNMHC5* showed that it contained many typical cis-acting elements, including those being significant for physiological activities: circadian rhythm (CAANNNNAC), phytohormones and sucrose signaling molecules (Table S1).

### 2.2. Subcellular Localization of GmNMHC5

**Figure 3:**
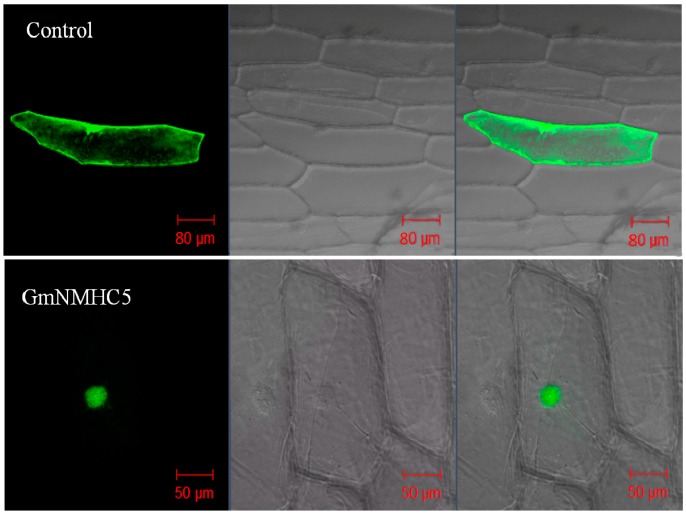
Cellular localization of eGFP and GmNMHC5-GFP fusion proteins. Photographs were taken in a dark field for green fluorescence (**left** column) and a bright field for cell morphology (**middle** column). The **right** column is the overlapping view of the dark and bright fields for comparative clarity.

To determine the subcellular localization of GmNMHC5, a transient expression assay of the fused protein (GmNMHC5-GFP) was carried out. Confocal microscopy observations suggested that the GmNMHC5-GFP fusion protein was located in the nucleus, strongly indicating its characteristic of being a transcription factor. In contrast, the free GFP protein in the control group was dispersed throughout the entire cell (Figure 3).

### 2.3. Expression Analysis of GmNMHC5

**Figure 4:**
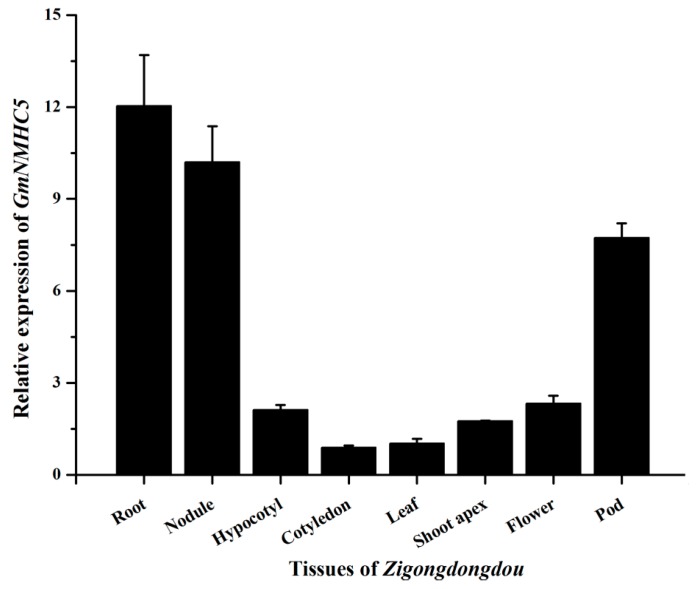
Tissue expression pattern of *GmNMHC5* revealed by real-time quantitative PCR at 29 days after SD treatment (DAT). The relative expression levels are normalized to *GmCYP2*. The data represent the mean ± SD of three independent experiments.

A real-time fluorescence quantitative PCR analysis was performed to investigate the expression of GmNMHC5 in different organs (root, nodules, hypocotyl, cotyledon, leaf, shoot apex, flower and pod). *GmNMHC5* was expressed in all the investigated plant tissues of Zigongdongdou under SD, however, an extremely high level of expression was found in the roots and nodules. *GmNMHC5* was also expressed at relatively high levels in pods though not as high as that in the roots or nodules (Figure 4).

**Figure 5:**
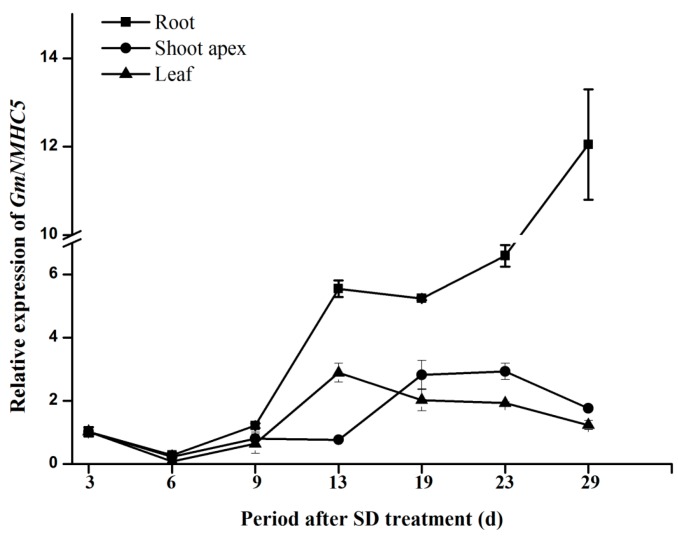
Expression analysis of *GmNMHC5* in different tissues under SD during the growth period. The relative expression levels are normalized to *GmCYP2*. The data represent the mean ± SD of three independent experiments.

A marked fluctuation in the expression levels of the gene was detected in the leaves, roots and shoot apexes under different days after SD treatment (DAT). The transcription level was observed to be maintained at a quite low level in the early vegetative growth period, and transcript started to accumulate in the late vegetative growth period. Interestingly, expression activation was first examined in leaves and roots (13 DAT), followed by shoot apexes (19 DAT) (Figure 5), showing a strict order of *GmNMHC5* expression in different tissues.

**Figure 6:**
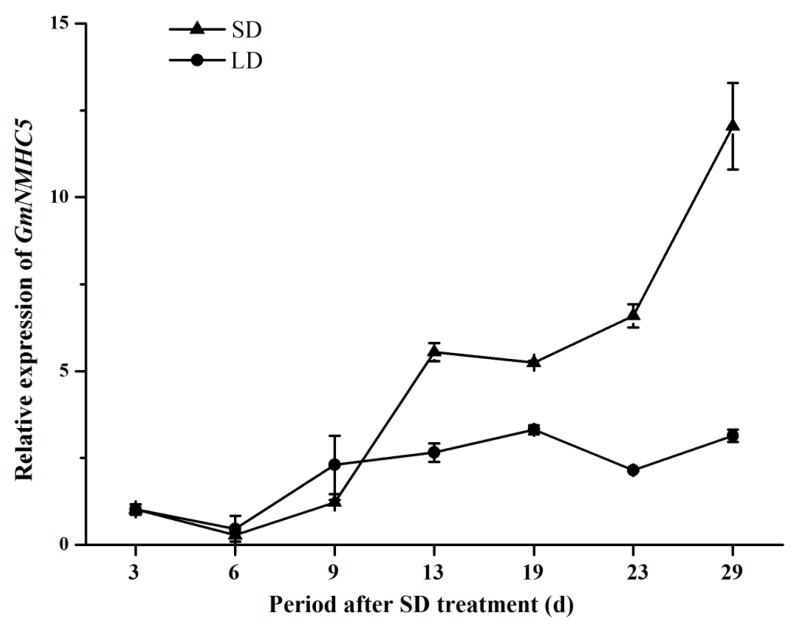
Expression analysis of *GmNMHC5* in roots under different photoperiod treatment. Real-time quantitative PCR analysis of *GmNMHC5* in roots at all growth periods under SD and LD, respectively. The relative expression levels are normalized to *GmCYP2*. The data represent the mean ± SD of three independent experiments.

Zigongdongdou is a photoperiod-sensitive late-flowering variety that only flowers under SD condition. In the present study, the *GmNMHC5* transcription levels in the roots of plants grown under LD (seedlings stay in the vegetative growth phase) and SD (seedlings can transform from vegetative growth to reproductive growth) were also quantified using real-time fluorescence quantitative PCR. No significant difference in *GmNMHC5* transcription under different light treatments was found before nine DAT. However, under SD, the *GmNMHC5* expression levels began to rise at 13 DAT, rose sharply once the plants transformed to the reproductive growth stage (23 DAT) and then peaked at the pod-setting stage (29 DAT). The levels also rose at nine DAT but increased in a more gentle way in non-podding soybeans under LD (Figure 6).

### 2.4. Sucrose Upregulates GmNMHC5

**Figure 7:**
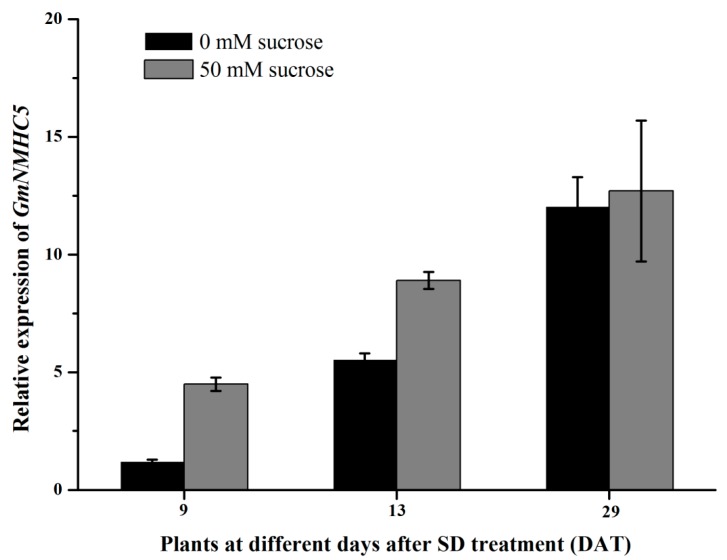
Expression of GmNMHC5 in the root of Zigongdongdou under sucrose treatment at different stage. The *X*-axis represents the nine days after SD treatment plants (9 DAT), the 13 days after SD treatment plants (13 DAT) and the 29 days after SD treatment plants (29 DAT).The relative expression levels are normalized to *GmCYP2*. The data represent the mean ± SD of three independent experiments.

The expression levels of GmNMHC5 in roots at different stages were determined 1 h after sucrose treatment. *GmNMHC5* transcript levels were increased with the exogenous sucrose application when the plants suffered in SD for 9 days, 13 days and 29 days (Figure 7), suggesting that a relative high sucrose level might be a signal for GmNMHC5 induction at three typical stages: before and at the floral meristem initiation stage and the podding stage. However, the gene expression pattern showed that it is less sensitive to sucrose at the podding stage compared to the floral meristem initiation stage.

### 2.5. GmNMHC5 Promotes Lateral Root Development

**Figure 8:**
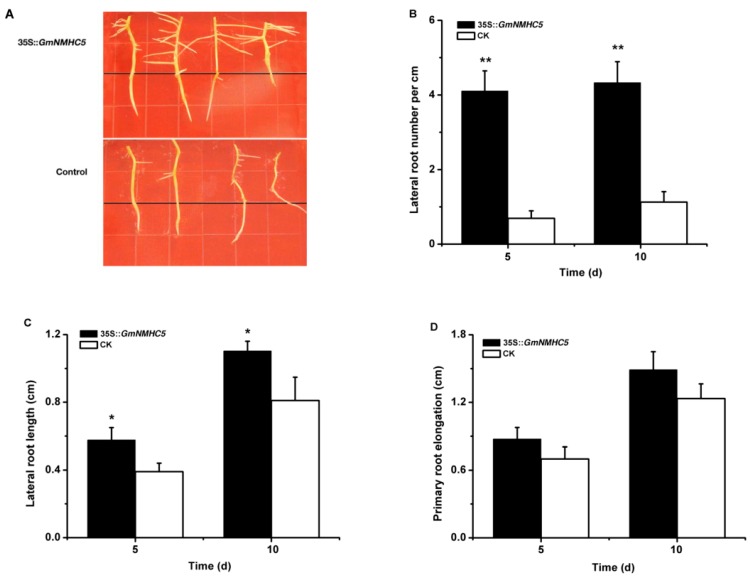
Overexpression of *GmNMHC5* promoted the growth of lateral roots. (**A**) Hairy roots of the plants transformed by pGUS-*GmNMHC5* or pGFPGUS*Plus* (as a control) via *A. rhizogenes* K599 with the binary vector, followed by 10 days of cultivation on 1/2 MS medium. After 5 days or 10 days of cultivation, the number of lateral roots from 30 transgenic hairy roots (**B**), the length of each longest lateral root from 30 transgenic hairy roots (**C**) and the elongation of 30 transgenic primary roots were recorded (**D**). The data represents the mean ± SD of three independent experiments. Means that are significantly different at the 1% (**) or 5% (*) confidence level, as detected by Student’s *t*-tests, are also shown.

To evaluate the effect of GmNMHC5 on lateral root development, the vector 35S::*GmNMHC5* was introduced into Zigongdongdou hairy roots using *A. rhizogenes*-mediated transformation. The vector pGFPGUS*Plus* was used as a control. The number and average length of lateral roots originating in the 35S::*GMNMHC5*-transformed hairy roots and the control roots were measured after five days and 10 days, respectively. The 35S::*GmNMHC5-*transformedhairy roots formed lateral roots that were both increased in number (4.1 *vs.* 0.8 per cm of the primary roots after 5 days and 4.2 *vs.* 1.2 per cm after 10 days) and length (0.58 *vs.* 0.38 cm after 5 days and 1.12 *vs.* 0.91 cm after 10 days) compared with the control roots when detected at two time points (Figure 8A–C). However, primary root elongation showed no significant differences between them (Figure 8D). These data indicate that GmNMHC5 promoted the development of the lateral root rather than the primary root.

### 2.6. GmNMHC5 Promotes Nodulation

**Figure 9:**
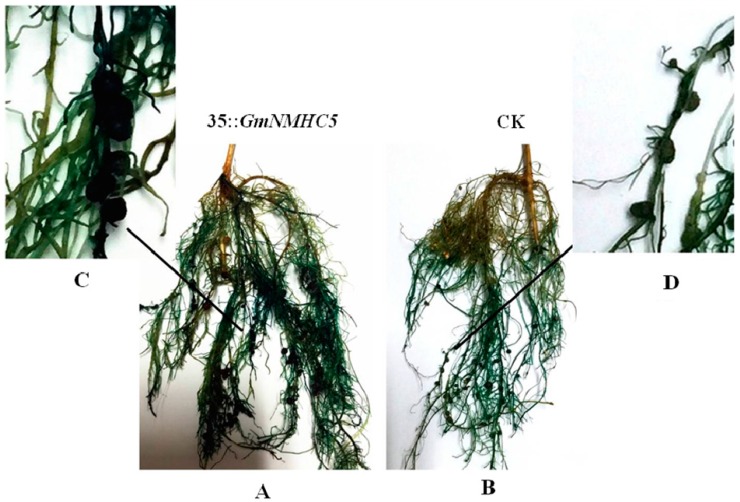
Gus staining of hairy roots transformed by *pGUS-GmNMHC5* and *pGFPGUSPlus* via *A. rhizogenes* K599 with the binary vector, (**A**) 35S::*GMNMHC5*-transformed hairy roots; (**B**) 35S::pGFPGUS*Plus*-transformed roots (control); (**C**) A segment of 35S::*GMNMHC5*-transformed hairy roots; (**D**) A segment of control roots.

**Figure 10:**
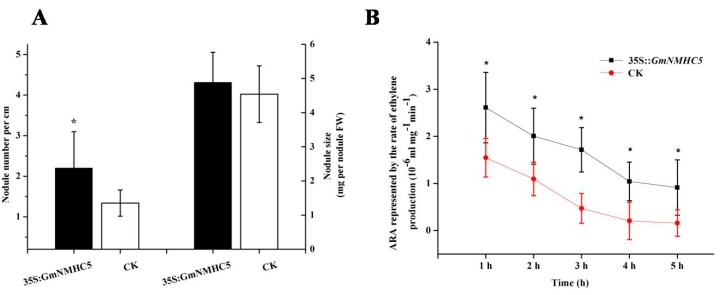
Overexpression of GmNMHC5 promoted nodulation and nodule nitrogen fixation activity. The number of nodules from transgenic hairy roots of 30 plants and the fresh weight of 50 nodules randomly selected from the transgenic hairy roots were recorded (**A**); the Acetylene reduction activity (ARA) represented by the rate of ethylene production with the extension of reaction time in the transgenic nodules and the control ones were measured (**B**); 40 nodules randomly selected from the Gus-positive hairy roots of eight plants were used in this assay. The data represents the mean ± SD of three independent experiments. Means that are significantly different at the 5% (*) confidence level, as detected by Student’s *t*-tests, are also shown.

Given the high expression levels of *GmNMHC5* in roots and its effects on promoting lateral root formation and development, its function in nodulation was further investigated. We overexpressed the gene by introducing 35S::*GmNMHC5* into roots via *A. rhizogenes-*mediated transformation and then inoculated the transgenic roots with *B. japonicum* USDA110. The vector pGFPGUS*Plus* was used as a control. After 20 days of cultivation, the average number of nodules on the 35S::*GMNMHC5*-transformed hairy roots (2.2 nodules per cm) was much higher than that on the control roots (1.34 nodules per cm) (Figure 9 and Figure 10). However, the size of the nodules was similar.

The nitrogenase activity of the nodules *in vitro* was measured by acetylene reduction assay. The nitrogenase initial rate was higher in the transgenic nodules, almost 1.7-fold that of control at 1 h. It declined with the extension of reaction time both in the transgenic nodules and the control ones, however, the former was always higher than the latter, suggesting that GmNMHC5 can promote the nodule nitrogen fixation activity (Figure 9D).

## 3. Discussion

### 3.1. Overexpression of GmNMHC5 Promotes Lateral Roots of Hairy Root Growth and Nodulation in Soybean

GmNMHC5 possesses the typical structural features of a MADS-box protein [17], including a strongly conserved MEF2-like MADS domain and the conserved K-domain, which has been shown to be important for protein-protein interactions [25]. The transient expression of the gene showed that the protein was localized to the nucleus of onion epidermal cells, which is consistent with the necessary feature of a transcriptional factor. The phylogenetic analysis of GmNMHC5 suggested that it belongs to the AGL17 subfamily. Many reports have confirmed that AGL17 subfamily members have significant roles in root system architecture construction [8,9,26,27]. The high expression level of *GmNMHC5* in the roots and nodules indicates that it might also be involved in root architecture construction (Figure 4). This hypothesis was further confirmed by the fact that *GmNMHC5-*transformed soybean developed significantly more lateral roots and nodules than the control roots (Figure 8, Figure 9 and Figure 10A). Moreover, the transformed nodules had higher nitrogen fixation activity (Figure 10B).

### 3.2. Expression of GmNMHC5 Is Induced in Floral Initiation and the Podding Period

Firstly, the real-time fluorescence quantitative PCR analysis of *GmNMHC5* transcription under SD suggested that mRNA levels in the leaves and roots began to accumulate at 13 DAT, whereas that in the shoot apexes began to accumulate at 19 DAT (Figure 5). Our previous study had proven that flower-bud differentiation in the apical meristem of Zigongdongdou under SD occurred exactly at the 13 DAT [28]; Secondly, the expression of *GmNMHC5* in the roots was not affected by treatments of different light duration before 9 DAT. However, expression was sharply promoted when the floral meristem was initiated (13 DAT) especially at the pod-formation stage (29 DAT) when the plants were treated under SD. However, the changing of its expression level was much more gentle in non-podding plants under LD (Figure 6). As its expression pattern was very similar to the flowering activators in soybean [29], further study could be focused on its function on flowering.

As a typical nodulating crop, the processes of nodule formation, nitrogen fixation and flowering play important roles in the development of soybean. However, these processes are highly energy consuming and result in fierce competition in the material and energy demands in vegetative and reproductive organs. Therefore, investigating the bifunctional regulation of both flowering and nodulation (including root development) is very important for understanding the cooperative associations of the two processes in soybean. In this study, the overexpression phenotype have shown that this new MADS-box protein gene, *GmNMHC5*, from soybean nodules is functional in regulation of root development and nodulation, even in nitrogen fixation. A knockout mutant should be constructed to confirm its function on root development, and the plant transformation is also necessary for the mining function, such as its involvement of flowering and pod setting.

A striking phenomenon observed in this study was that the expression activation of *GmNMHC5* occurred first in the leaves and roots, followed by the shoot apexes, showing a strict order of expression profile in different tissues (Figure 5). Therefore, the transcription of *GmNMHC5* might be regulated by one or more signaling molecules during the development of soybean, especially in the transition from vegetative growth to reproductive growth. Recently, many researchers began to focus on the roles of phytohormones or sugar as signaling molecules to regulate gene expression and eventually development in plants [30,31,32,33,34,35,36]. In our study, *GmNMHC5* was upregulated by sucrose (Figure 7), the analysis of the upstream region of *GmNMHC5* revealed several hormone and sucrose-responsive elements (Table S1), indicating that the cooperative regulation of GmNMHC5 in root development/nodulation in soybean might occur through phytohormones and sucrose, though the actual functional relationship is under investigation in our lab.

The cooperative regulation of distant organs (e.g., the process of flowering occurs in the aerial parts, whereas nodulation occurs in the underground parts) in plants is very complex. This study revealed that GmNMHC5 can improve lateral root development and nodulation. This initial research on the function of GmNMHC5 should be followed by further work that focuses on the whole plant transfomation in order to thoroughly explore its function in flowering, and the crosstalk between this gene and signal molecules such as hormones and sucrose.

## 4. Experimental Section

### 4.1. Plant Materials and Growth Conditions

Zigongdongdou, a late-maturing and light-sensitive soybean (*Glycine max* [L.] Merr.) cultivar from Zigong, Sichuan Province in south China [37], was used for most of the experiments. Plants were grown in a controlled culture room at 24 °C with a relative humidity of 60% either under short day conditions (SD) (8 h light/16 h dark) or long day conditions (LD) (16 h light/8 h dark). For hairy root transformation, the seeds of Zigongdongdou were surface-sterilized for 16 h using chlorine gas produced by mixing 5 mL of 12 M HCl and 100 mL commercial bleach in a tightly sealed desiccator.

### 4.2. Cloning of the GmNMHC5 Gene

The Phytozome database (http://www.phytozome.net/) was used to search for the protein and then the coding sequence (CDS) with the highest similarity to *NMHC5* (GenBank accession No. AAB51377.1) in *Medicago sativa* (*MsNMHC5*). A specific pair of primers, *GmNMHC5*-primer1-forward and reverse (Table S2), were applied for the first round of PCR using Zigongdongdou nodule cDNA as a template. Those PCR products with the expected size were used as the template for the second round of nested amplification with a pair of primers, *GmNMHC5*-primer2-forward and *GmNMHC5*-primer2-reverse (Table S2). The amplified product of the *GmNMHC5* gene was sequenced; and the deduced protein sequence was aligned with MsNMHC5 and the production of other root-specific expression MADS-box genes using Clustal X2 (Conway Institute UCD, Dublin, Ireland). The conserved domains were determined using Conserved Domain Database (CDD) V.3.10 (http://www.ncbi.nlm.nih.gov/Structure/cdd/wrpsb.cgi). The genetic relationships between GmNMHC5 and the reported MIKC-type MADS-box transcription factors of soybean and Arabidopsis were established using the Maximum Likelihood method of phylogenetic tree construction, with 200 bootstrap replicates, using MEGA v.5.05 (Center for Evolutionary Medicine and Informatics, Tempe, AZ, USA). The number for each node is the bootstrap percentages, and nodes with less than 70% bootstrap values were collapsed.

### 4.3. Transient Expression of a GmNMHC5-GFP Fusion Protein in Onion Epidermal Cells

The open reading frame (ORF) of *GmNMHC5* was fused with the N-terminus of EGFP under the control of the CaMV 35S promoter. The *GmNMHC5* gene, amplified by PCR using a forward primer containing an XbaI site and a reverse primer containing a SalI site (Table S2: XbaI-*GmNMHC5* and SalI-*GmNMHC5*), was introduced into p16318-GFP for particle bombardment. Each plasmid DNA was constructed into vector-coated microprojectiles [38]. The inner epidermal layers of onion (*Allium cepa*) were placed on MS medium [39] with the inner-side up and cultivated for 5–6 h and then bombarded with the vector-coated microprojectiles using a PDS-1000/He hand-held gene gun (Helios, Bio-Rad, Hercules, CA, USA) with a 9 cm shot distance, 25 mHg vacuum and 1100 psi rupture disc pressure. After transformation, the onion layers were incubated for 16 h at 22 °C in the dark. GFP signals were analyzed using a confocal microscope Zeiss LSM710 (Carl Zeiss, Oberkochen, Germany).

### 4.4. Bioinformatic Analysis of the GmNMHC5 Promoter

The 2300-bp DNA sequence upstream of the start codon of *GmNMHC5* was obtained from the Phytozome database. The prediction of the cis-acting elements was conducted using online software: http://www.dna.affrc.go.jp/PLACE/signalup.html.

### 4.5. Construction of 35S::GmNMHC5 for Overexpression of the Gene

For overexpression of *GmNMHC5* in hairy roots, a 35S::*GmNMHC5*plasmid was constructed with *GmNMHC5* replacing the *egfp* gene in the pGFPGUS*Plus* vector, so that *GmNMHC5* can be driven by the CaMV 35S promoter. The pair of primer XbaI-*GmNMHC5* and SacI-*GmNMHC5* (Table S2) was used to amplify the *GmNMHC5* gene.

### 4.6. Expression Analysis of the GmNMHC5 Gene

To evaluate the expression pattern of *GmNMHC5*, soybean plants were grown in a controlled culture room under SD or LD. All The samples were sliced off and quickly frozen in liquid nitrogen. Total RNA was extracted from 100 mg of sample using the Trizol reagent (Invitrogen, Carlsbad, CA, USA). The first-strand cDNA was amplified from the total RNA with Superscript II reverse transcriptase (Invitrogen, CA, USA). Real-time quantitative PCR was performed using the ABI7900 (Applied Biosystems, Foster City, CA, USA) with the Takara SYBR Premix Extaq Kit (Takara, Kusatsu, Japan). The real-time quantitative PCR data were analyzed using SDS2.3 software (Applied Biosystems, Foster City, CA, USA). The primers used for real-time quantitative PCR of *GmNMHC5* (*GmNMHC5-*qRT-F; *GmNMHC5-*qRT-R) and the internal reference (CYP2-forward and CYP2-reverse) are listed in Table S2.

### 4.7. Sucrose Treatment

Initially, the soybean seeds were sowed in soil and grown under SD condition, then the seedlings were cultivated in Hoagland solution 5 days after germination. Plants subjected to SD for 9 days, 13 days and 29 days were cultivated in Hoagland solution supplement with 50 and 0 mM (as control) sucrose. Roots were collected after 1 h treatment and frozen in liquid nitrogen for RNA extraction.

### 4.8. Agrobacterium rhizogenes-Mediated Hairy Root Transformation

To verify the effects of GmNMHC5 on root development, *Agrobacterium rhizogenes*-mediated hairy root transformation was carried out *in vitro* according to the method described by Cao *et al.* [40]. A fresh culture of *A. rhizogenes* strain K599 containing 35S::*GmNMHC5* was introduced into Zigongdongdou hairy roots. The pGFPGUS*Plus* containing K599 strain was used as a control inoculant. When the hairy roots emerged at the wound sites, the transgenic roots were verified by GUS activity analysis. The GUS-positive roots were sliced into similar initial-length segments with the root tips and placed on 1/2 MS medium. The numbers and lengths of lateral roots and the elongation of the primary roots were measured after cultivation at 24 °C (relative humidity, 60%) for 5 days and 10 days, respectively.

For verifying the effects of GmNMHC5 on nodulation, Zigongdongdou seedlings with unfolded cotyledons were injected with K599 strain containing 35S::*GmNMHC5* by stabbing at their cotyledonary node several times with a syringe needle. The pGFPGUS*Plus* containing K599 strain was used as a control inoculant. The plants were transplanted into a pot with fresh wet vermiculite 7 days after the emergence of hairy roots and watered with 200 mL of Fahraeus nitrogen-free nutrient solution [41] and 20 mL of *Bradyrhizobium japonicum* USDA110 culture (*A*_600_ = 0.6) per pot every 7 days. For the assay of nodule number and size, the transgenic roots were confirmed by GUS activity after 20 days of cultivation, the number of nodules on the 35S::*GMNMHC5*-transformed hairy roots and that on the control roots was recorded, nodule size was calculated as the average fresh weight of a single nodule. For nitrogen fixation activity test, in order to ensure the activity of nodules, only the root segments were stained by GUS, nodules on the 35S::*GMNMHC5*-transformed hairy roots and the control roots were collected separately for the acetylene reduction assay. Forty nodules randomly selected from the Gus-positive hairy roots of 8 plants were placed in 70 mL glass vessels. Acetylene gas was introduced to a final concentration of 10% (*v*/*v*) and samples were incubated at 28 °C. Gas samples (0.2 mL) were extracted using a syringe and then injected into a gas chromatograph GC2010 to determine the amount of ethylene formed (mL C_2_H_2_ mg·nodules^−1^·min^−1^) every 1 h.

## 5. Conclusions

GmNMHC5 is an MIKC-type MADS-box transcription factor, and its sequence of amino acids showshigh similarity to the AGL17 subfamily in Arabidopsis. Its transcripts were more abundant in roots and nodules, and can be upregulated by exogenous sucrose. Overexpression of *GmNMHC5* in transgenic soybean significantly promoted lateral root development and nodulation. The expression levels of GmNMHC5 were increased at the floral meristem initiation and pod setting stage under short-day conditions. Further study is needed to explore the function of GmNMHC5 in flowering and the pod setting, as well as the crosstalk between sucrose or other hormones and gene expression and function.

## Supplementary Materials

Supplementary materials can be found at http://www.mdpi.com/1422-0067/16/09/20657/s1.
