# Supplementary material for: A Novel Sucrose-Regulatory MADS-Box Transcription Factor GmNMHC5 Promotes Root Development and Nodulation in Soybean (Glycine max [L.] Merr.)
Source: Int J Mol Sci. 2015 Aug 31;16(9):20657–73. doi: 10.3390/ijms160920657 (PMC4613224; doi:10.3390/ijms160920657)
Supplement: Supplementary file 1 [file ijms-16-20657-s001.pdf]

Supplementary Information

Table S1. Typical cis-acting elements in the region upstream of *GmNMHC5*by PLACE.

Element Name	Sequence	Function of Site	Position from Translation Start Site	Frequency
ABRELATERD1	ACGTG	ABA-responsive element	−606; −744	2
ARR1AT	NGATT	ARR1-binding element/Cytokinin-responsive element	−1342; −1499; −1651; −1784; −1857...	16
CATATGGMSAUR	CATATG	Auxin-responsive element	−729; −1264	2
GAREAT	TAACAAR	GA-responsive element	−1511	1
GT1CONSENSUS	GRWAAW	Binding site for light-regulated genes	−776; −1015; −1441; −1708; −1797; −1841...	10
NODCON1GM	AAAGAT	Putative nodulin consensus sequence	−1662; −1789; −1893	3
NODCON2GM/	CTCTT	Putative nodulin consensus sequence	−95; −283	2
OSE2ROOTNODULE	CTCTT	Consensus sequence motifs of the promoters in infected cells of nodules	−95; −283	2
REALPHALGLHCB21	AACCAA	Required for phytochrome regulation	−2018	1
REBETALGLHCB21	CGGATA	Required for phytochrome regulation	−853	1
RHERPATEXPA7	KCACGW	Root hair-specific element	−638; −84	2
SURE1STPAT21	AATAGAAAA	Sucrose-responsive element	−1708	1
WRKY71OS	TGAC	Binding site of a transcriptional repressor of the GA signaling pathway	−234; −317; −414; −807; −1293; −1853...	8

N indicates A, C, G or T; W indicates A or T; R indicates A or G; … indicates *etc*.

Table S2. Sequences of primers used in this study.

Primer Name	Primer Sequence
**For cloning *GmNMHC5***	
GmNMHC5-primer1-forward	5′-TATAATTTTTTGACCAAAGAT-3′
GmNMHC5-primer1-reverse	5′-GCACATATGACAACCTCCCTGATTACTG-3′
GmNMHC5-primer2-forward	5′-ATGGGGAGAGGTAAGATTGCGATTC-3′
GmNMHC5-primer2-reverse	5′-CTAATGCAGGTGCAATCCGAGTTTC-3′
**For Construction of 35S::*GmNMHC5-GFP* and 35S::*GmNMHC5***	
XbaI-*GmNMHC5*	5′-GCTCTAGAATGGGGAGAGGTAAGATTGC-3′
SalI-*GmNMHC5*	5′-ACGCGTCGACCTAATGCAGGTGCAATCCGAGT-3′
SacI-*GmNMHC5*	5′-CGAGCTCCTAATGCAGGTGCAATCCGAGTTTC-3′
**For Real-Time Quantitative PCR**	
GmNMHC5-qRT-F	5′-GCGGTTATTGAACGCTACAACA-3′
GmNMHC5-qRT-R	5′-CTTGCTGCTTCTGTCTGCCA-3′
CYP2-forward	5′-CGGGACCAGT GTGCTTCTTCA-3′
CYP2-reverse	5′-CCCCTCCACT ACAAAGGCTCG-3′
